# CLDN18.2: a potential nanotherapeutic target for cholangiocarcinoma

**DOI:** 10.3389/fphar.2025.1559558

**Published:** 2025-03-26

**Authors:** Yu Huang, Yulu Ye, Tingzhuang Yi, Cheng Yuan, Daojun Li

**Affiliations:** ^1^ Department of Oncology, Yichang Central People’s Hospital and The First College of Clinical Medical Science, China Three Gorges University, Yichang, Hubei, China; ^2^ Clinical Medical College, YouJiang Medical University for Nationalities, Baise, Guangxi, China; ^3^ Department of Oncology, Affiliated Hospital of YouJiang Medical University for Nationalities/Guangxi Clinical Medical Research Center for Hepatobiliary Diseases, Baise, Guangxi, China; ^4^ Tumor Prevention and Treatment Center of Three Gorges University and Cancer Research Institute of Three Gorges University, Yichang, Hubei, China; ^5^ Clinical Medical Research Center for Precision Diagnosis and Treatment of Lung Cancer and Management of Advanced Cancer Pain of Hubei Province, Wuhan, China

**Keywords:** nanotherapeutic target, CLDN18.2, bispecific antibodies (BsAbs), antibody-drug conjugate (ADC), zolbetuximab, chimeric antigen receptor T (CAR-T), cholangiocarcinoma (CCA)

## Abstract

Cholangiocarcinoma (CCA) is an extremely malignant and aggressive primary liver tumor that has become increasingly prevalent in recent years. Unfortunately, the prognosis for patients diagnosed with CCA remains exceptionally poor. Currently, the primary treatment options include surgery and chemotherapy. However, the effectiveness of postoperative chemotherapy is limited, characterized by a brief duration of remission and high rates of recurrence and metastasis, resulting in minimal survival benefits for patients. Therefore, there is an urgent need to develop new therapeutic strategies that are both safer and more effective. In recent years, as oncology research has progressed, Claudin 18.2 (CLDN18.2)-targeted therapy has emerged, showing promise for improving the survival of patients with CLDN18.2-positive cancers. Studies suggest that combining new agents targeting CLDN18.2 with standard cytotoxic therapies offers significant survival benefits in CLDN18.2-positive solid tumors, which is expected to provide a more effective treatment option for patients with advanced cholangiocarcinoma. While existing immune checkpoints or therapeutic targets have limitations, such as low positivity rates and minimal absolute improvement in patient survival time, drugs that target FGFR, IDH, and Her-2, along with antiangiogenic agents, have shown promise for patients with advanced malignancies affecting the bile ducts. Therefore, exploring these novel therapeutic strategies may yield new insights for precision treatment of cholangiocarcinoma in the future. This review aims to focus on the potential application of CLDN18.2 in treating solid tumors, particularly cholangiocarcinoma, to systematically summarize research progress related to this target and thoroughly examine its value in diagnosing, treating, and assessing the prognosis of cholangiocarcinoma.

## 1 Introduction

Cholangiocarcinoma (CCA) refers to a group of malignant epithelial tumors that develop in different areas of the biliary tree, characterized by the differentiation of cholangiocarcinoma cells (Sekiya and Suzuki, 2012). These cancer cells may arise from cholangiocarcinoma, peribiliary glandular, or hepatic progenitor cells (Fan et al., 2012). CCA is known for its high level of aggressiveness. Clinically, CCA is typically classified by the anatomical location of the biliary tract tumors into intrahepatic cholangiocarcinoma (iCCA) and extrahepatic cholangiocarcinoma (eCCA), with the secondary bile ducts serving as the dividing line. The latter category is divided into portal cholangiocarcinoma (pCCA) and distal cholangiocarcinoma (dCCA) (Chmiel et al., 2022). Most cases of CCAs are primary, with long-term irritation of the biliary epithelium and chronic inflammation as the main high-risk factors (Chmiel et al., 2022). A few instances arise from other conditions, including cirrhosis, viral hepatitis, primary sclerosing cholangitis, and cystic diseases of the bile ducts, such as congenital cystic dilatation of the intrahepatic bile ducts, along with hepatic schistosomiasis, cholelithiasis, and choledocholithiasis, among others (Razumilava and Gores, 2014; Plentz and Malek, 2015; Tyson and El-Serag, 2011; Gupta and Dixon, 2017). In the early stages, CCA typically shows no noticeable symptoms, while advanced stages present common signs such as painless progressive jaundice, abdominal pain, nausea, vomiting, and weight loss (Chmiel et al., 2022).

As a relatively rare and highly diverse tumor, CCA exhibits significant regional variations in incidence. Its occurrence is low in most European and American countries but higher in certain Southeast Asian regions, such as China, South Korea, and Thailand (Florio et al., 2020). The rate of eCCA typically remains lower than that of iCCA in most European and American countries (Qurashi et al., 2025). In contrast, in China, the situation is reversed; eCCA accounts for approximately 80%–90%, while iCCA makes up less than 10% (Chmiel et al., 2022). Global mortality rates for iCCA and eCCA show distinct epidemiological patterns, with iCCA demonstrating greater lethality than eCCA in most countries (Qurashi et al., 2025). In recent years, the incidence of CCA has shown a consistent upward trend (Banales et al., 2020). According to the latest statistics from the International Agency for Research on Cancer (IARC), intrahepatic cholangiocarcinoma accounts for 15% (Khan et al., 2019), ranking second to hepatocellular carcinoma. The global age-standardized incidence rate of CCA is approximately 165.5 cases per 100,000 people, with Shanghai, China, reporting 7.6 cases per 100,000 people (Qurashi et al., 2025; Khan et al., 2019). Despite its low incidence rate, cholangiocarcinoma is highly invasive, leading to most patients being diagnosed at advanced stages and typically succumbing within 1 year of their diagnosis (Liu et al., 2021). According to the latest statistics, the median overall survival for cholangiocarcinoma is only 8.8 months (Wang et al., 2024). The long-term prognosis for CCA is inferior, with a 5-year survival rate of just 7%–20% (Chmiel et al., 2022; Merters and Lamarca, 2023; Pirozzi and Yan, 2021), significantly lower than the rates of other common malignant tumors, such as prostate cancer (70%–100%), breast cancer (80%–85%), colorectal cancer and cervical cancer (50%–70%) (Lippi and Mattiuzzi, 2019).

In recent years, the primary treatment options for cholangiocarcinoma have included surgery, chemotherapy combined with immunotherapy, molecularly targeted therapy, ablation therapy, and radiation therapy (Brindley et al., 2021; Moris et al., 2022). Approximately 20%–30% of patients with cholangiocarcinoma can undergo early curative surgical resection; however, the 5-year survival rate following surgery remains only 20%–35% (Moris et al., 2022). For patients with locally advanced, unresectable, or metastatic cholangiocarcinoma, the currently recommended first-line treatment regimens involve chemotherapy combined with immunotherapy (Moris et al., 2022; Ilyas et al., 2017), leading to a median overall survival (mOS) of 11.1–11.7 months with chemotherapy alone (gemcitabine + cisplatin) and 14 months when combined with programmed death receptor-1 (PD-1) inhibitors (Brindley et al., 2021). Nonetheless, the efficacy remains limited. Emerging molecularly targeted drug therapies have significantly impacted second-line treatment for cholangiocarcinoma (Harding et al., 2023). Approximately 20% of cholangiocarcinoma patients qualify for targeted therapies that involve specific genes, including FGFR, IDH, HER-2 (ERBB2), RAS-RAF-MEK (MAP2K1)-ERK (MAPK3), NTRK, RET, and BRAF (Dieci et al., 2013; Goyal et al., 2021; Lee et al., 2021; Saha et al., 2016; Kam et al., 2021; Kelley et al., 2020). However, these targeted therapies demonstrate minimal effectiveness and have not yet substantially improved patient prognosis. The lack of effective diagnostic methods in the early stages, along with insufficient treatments during late-stage cholangiocarcinoma, poses significant challenges for clinical management. Therefore, there is an urgent need to explore new therapeutic targets to transform the current treatment landscape, ultimately aiming to enhance patients’ prognosis and quality of life.

Claudin proteins are essential components of tight junctions (TJs) that are crucial in forming intercellular adhesion structures. Their isoform, claudin18.2 (CLDN18.2), is specifically expressed in differentiated gastric epithelial cells and is excessively activated during cellular carcinogenesis. This isoform is widely and aberrantly expressed in various gastric and gastroesophageal junction (GC/GEJ) adenocarcinomas and esophageal, pancreatic, and colorectal solid tumors. In recent years, several innovative drugs targeting CLDN18.2 have achieved significant breakthroughs in treating advanced CLDN18.2-positive cancers, offering new hope to many patients with CLDN18.2-positive tumors. This article aims to summarize and discuss the considerable advancements in the treatment of solid tumors and the potential applications of CLDN18.2 in diagnosing, treating, and assessing the prognosis of cholangiocarcinoma.

## 2 Claudin18.2 (CLDN18.2)

**FIGURE 1:**
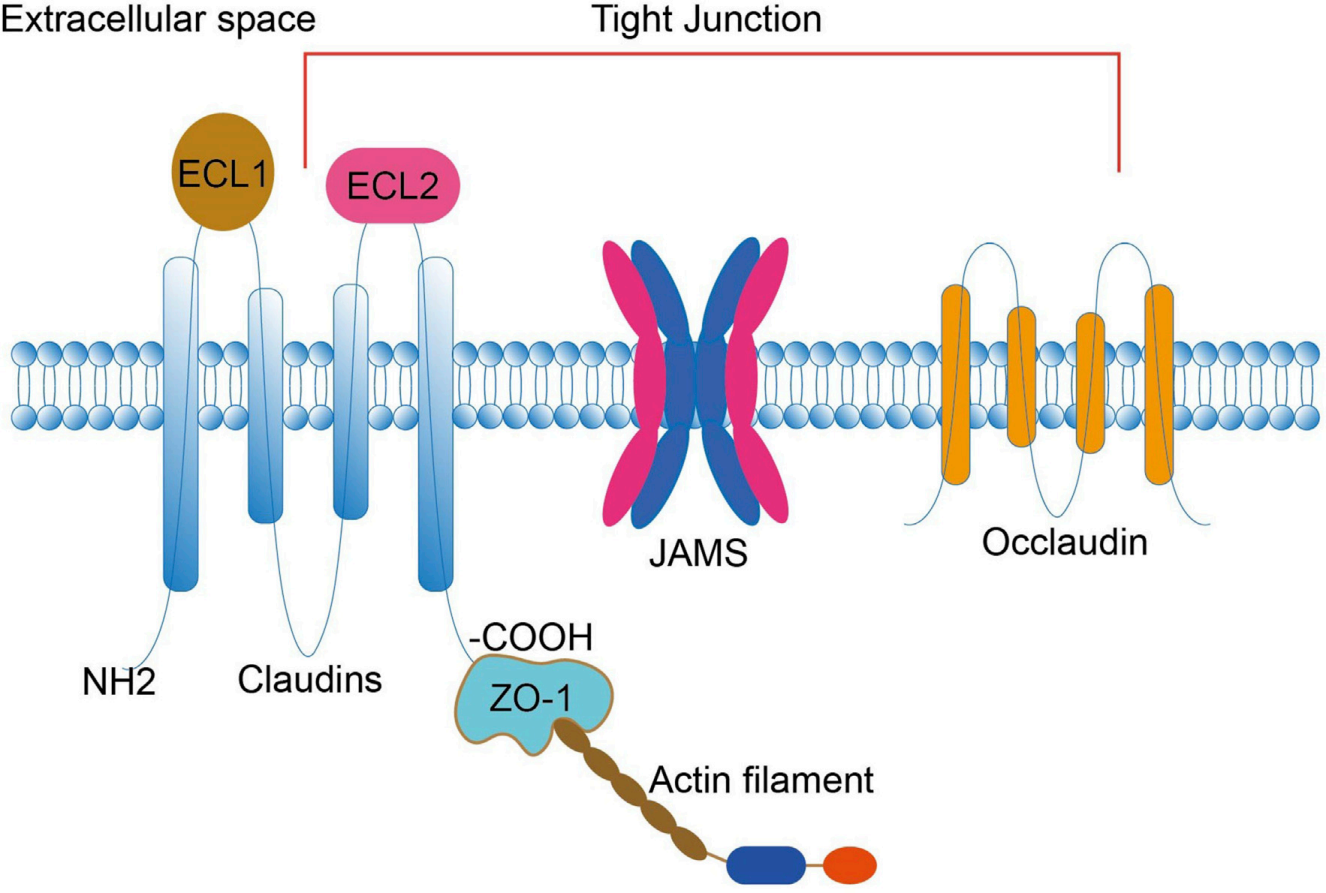
Claudins consist of four transmembrane structural domains: an N-terminal cytoplasmic region, two extracellular loops (ECL1 and ECL2), an intracellular loop, and a C-terminal cytoplasmic region. The C-terminus interacts with the ZO-1 scaffold protein, attaches CLDN to actin fibers, and recruits other tight junction proteins. ECL1 contains four β-strands and an extracellular helix (ECH), and ECL2 contains a β-strand and a cell-surface-exposed part of the transmembrane structural domain. Claudin, together with JAM and occludin, constitutes a TJ. TJs are located within epithelial and endothelial cells and have roles in maintaining intercellular adhesion, cell polarity, and paracellular permeability. ECL, extracellular loop; JAM, junctional adhesion molecule; TJ, tight junction; ZO, Zona Occludens.

Claudins are crucial components of tight junctions (Günzel and Yu, 2013), a type of integrin membrane protein encoded by the CLDN gene, which forms intercellular adhesion structures. The mammalian CLDN gene family consists of 27 family members. Claudins are found in epithelial and endothelial tight junctions, and their four transmembrane helices play a vital role in maintaining the polarity of epithelial and endothelial cells. This structure constitutes both a paracellular channel and a paracellular barrier. It maintains intercellular adhesion, cell polarity, and paracellular permeability through coordinated actions with other transmembrane proteins of tight junctions and cytoplasmic scaffolding proteins (Günzel and Yu, 2013; Chen et al., 2013; Gowrikumar et al., 2019) (Figure 1). Changes in the expression of Claudins may lead to impaired tight junction function, which affects the signaling pathway and acts as a tumor-promoting event in some epithelial cancers (Sugimoto and Chiba, 2021). Although Claudins are widely expressed in different tissues, not all are present in all tissues simultaneously. Different tissues express different claudin proteins; the same tissue may express multiple claudin proteins (Koval, 2013). Furthermore, other tissues may also express the same claudin protein (Liu et al., 2016). The expression level is tissue heterogeneous, and the variability in the expression of these claudins regulates cellular function and confers unique properties to paracellular barrier function.

Claudin18.2 (CLDN18.2) is a variant of the transmembrane protein claudin18 encoded by the CLDN18 gene (Türeci et al., 2011). The CLDN18 gene has two distinct exons (1a and 1b) that undergo transcription and splicing to produce two isoforms, CLDN18.1 and CLDN18.2 (Türeci et al., 2011; Liu et al., 2022). Under normal conditions, these isoforms correspond to the tight junction proteins claudin-18.1 and claudin-18.2, specific to lung and gastric tissues, respectively. CLDN18.2 is a particular protein found in the tight junctions of the gastric mucosa (Sahin et al., 2008). It is present only in the specialized epithelial cells of the stomach lining (Sahin et al., 2008). This protein is not found in gastric stem cells and plays a role in maintaining barrier function and protecting against gastric acid (Chen et al., 2023). In malignant transformation cases, such as gastric carcinoma development, tight junctions are disrupted, and cell polarity changes, which exposes the CLDN18.2 protein on the cell surface (Sahin et al., 2008; Sahin et al., 2021). CLDN18.2 is expressed not only in gastric adenocarcinoma (Coati et al., 2019; Jun et al., 2014) but also in various solid tumors, such as gastroesophageal junction (GEJ), pancreatic, colon, nonsmall cell lung, breast, head and neck, and bronchial cancers (Sahin et al., 2008; Yan et al., 2024; Cao et al., 2022). Studies have shown that CLDN18.2 can be either upregulated or downregulated in these tumors and that these changes may contribute to the development and progression of tumors (Sahin et al., 2008; Coati et al., 2019; Jun et al., 2014). In non-metastatic gastric adenocarcinomas, the expression of CLDN18.2 exhibited heterogeneity in both upregulation and downregulation. In contrast, metastatic gastric adenocarcinomas and other solid tumors generally showed upregulation of CLDN18.2 expression. These findings suggest that CLDN18.2 may play a role in tumor cell proliferation, differentiation, and distant metastasis and could be an independent predictor of poor tumor prognosis.

## 3 Diagnosing and treating cholangiocarcinoma can be challenging

Cholangiocarcinoma is a highly lethal malignant tumor of the digestive tract. It has an insidious onset and no apparent symptoms in the early stages. Most patients have already progressed to locally advanced or distant metastatic disease by the time they develop symptoms or are diagnosed for the first time. This characteristic dramatically increases treatment difficulty in the later stages and results in unsatisfactory treatment efficacy. Additionally, CCA is prone to recurrence, resulting in a poor prognosis for patients. At the molecular level, many proteins and cytokines are considered characteristic molecular biomarkers in genetic research and are applied in cholangiocarcinoma. These biomarkers can be used for early detection, screening, diagnosis, and staging, monitoring therapeutic efficacy, judging prognosis, and monitoring the recurrence of bile duct cancer. They also serve as differential indices for identifying the primary foci of metastatic tumors of unknown origin and for evaluating patient prognosis. Furthermore, they monitor patient prognosis and tumor response to different treatment modalities. However, the biomarkers currently available for evaluating the effectiveness of CCA treatment and predicting disease progression lack the necessary specificity and sensitivity. For example, the most valuable molecular marker, CA19-9, is not sufficiently specific for patients diagnosed with CCA for the first time because it is also abnormally elevated in other solid tumors. At the genetic level, low positivity rates for existing therapeutic targets and insignificant improvements in survival are limitations. Although the overall gene variant rate of CCA has been reported to be approximately 40%, drugs that target CCA gene variants have become second-line clinical treatments, with FGFR2 fusion and IDH gene mutations as the main targets of action. However, the positive rate of single-gene therapeutic targets is low. Many studies have shown that patients with cholangiocarcinoma (CAA) who have access to targeted drug therapy are more likely to have intrahepatic cholangiocarcinoma (ICC). In contrast, limited data show a clinical benefit for patients with extrahepatic cholangiocarcinoma (ECC). In clinical practice, very few cholangiocarcinoma patients who have undergone genetic testing for therapeutic targets related to cholangiocarcinoma have tested positive, and even fewer have the opportunity to receive targeted drug therapy.

The lack of accurate diagnostic methods for CCA in the early stages and effective therapeutic means in the late stages has posed a significant challenge in its clinical management. Therefore, a deeper understanding of CCA is crucial for improving its diagnosis and treatment outcomes. New biomarkers for the diagnosis and prognosis of CCA, as well as new effective therapeutic targets, urgently need to be identified. This information will help improve the diagnosis and treatment of cholangiocarcinoma, increase the survival rate, and ultimately improve the prognosis of patients.

### 3.1 Cholangiocarcinoma and molecular markers

At this stage, biomarkers available for the assessment of CCA include glycan antigen 19-9 (CA19-9) (Razumilava and Gores, 2014), carcinoembryonic antigen (CEA) (Uenishi et al., 2007), Dickkopf-related protein 1 (DKK1) (Rim et al., 2022; Shi et al., 2012; Jarman et al., 2022), serum cytokeratin 19 fragment (CYFRA21-1) (Zeng et al., 2018; Nakamura et al., 2019), fibroblast growth factor receptor (FGFR) (Goyal et al., 2021; Vogel et al., 2023), and isocitrate dehydrogenase isoenzyme (IDH) (Wu et al., 2022) (Table 1).

**TABLE 1:** Some biomarkers associated with cholangiocarcinoma.

Biomarkers	Optimal threshold	Sensitivity	Specificity	Superiority	Deficiency	Tumor correlation
CA19-9	3.7 ng/mL	62.0%	92.2%	Monitoring efficacy and recurrence of cholangiocarcinoma	Limited specificity and sensitivity	Expression levels positively correlated with poor tumor prognosis.
CEA	39.0u/mL	54.9%	87.8%	As a reference indicator for review	Poor specificity and sensitivity	_
DKK1	2.49 ng/mL	75.7%	100%	Good specificity	Poor sensitivity; only for intrahepatic cholangiocarcinoma.	Expression levels positively correlated with poor tumor prognosis.
CYFRA21-1	2.7 ng/mL	74.7%	92.2%	As a prognostic monitor for tumors	Limited specificity and sensitivity; only for intrahepatic cholangiocarcinoma.	Expression levels positively correlated with poor tumor prognosis.
IDH	—	—	—	—	—	—
FGFR2	—	—	—	—	—	—

Aberrantly expressed biomarkers in cholangiocarcinoma include CEA, CA19-9, DKK1, CYFRA21-1, IDH, and FGFR2. CA19-9, DKK1, and CYFRA21-1 are positively correlated with the poor prognosis of cholangiocarcinoma and can be used to indicate efficacy and recurrence monitoring in cholangiocarcinoma. IDH and FGFR2 are primarily used as drug therapy targets for gene variants in cholangiocarcinoma. CA19-9, glycan antigen 19-9; CEA, carcinoembryonic antigen; DKK1, Dickkopf-related protein 1; CYFRA21-1, serum cytokeratin 19 fragment.

CA19-9 is a glycoprotein that combines salivary glycolipids and salivary glycoproteins. CEA is a structural protein in the membrane of cancer cells, and both CA19-9 and CEA are commonly used as tumor markers in clinical practice. The optimal cutoff value for CEA is 3.7 ng/mL (sensitivity, 54.9%; specificity, 87.8%), which is not sufficiently sensitive to support the diagnosis of ICC (Uenishi et al., 2007). The optimal cutoff value for CA19-9 is 39.0 U/mL (sensitivity, 62.0%; specificity, 92.2%), which is considered a sensitive serological marker for the diagnosis and follow-up of ICC but lacks absolute specificity (Uenishi et al., 2007). CEA and CA19-9, which are commonly expressed in various tumors, do not have advantages in terms of specificity and sensitivity over CCA and are not ideal tumor-specific molecular markers. DKK1 is a secreted modulator of WNT signaling (Rim et al., 2022) that acts as an immunomodulator *in vivo* and can be expressed in ICC tumor tissues and serum to promote iCCA tumor immune escape by recruiting immunosuppressive macrophages (Jarman et al., 2022). The optimal serum threshold for DKK1 in ICC is 2.49 ng/mL (sensitivity SE, 75.7%; specificity SP, 100%), with approximately 38.4% of patients exhibiting positive DKK1 expression (Shi et al., 2012). DKK1 promotes ICC lymph node metastasis by enhancing ICC tumor cell invasion, and its expression level is positively correlated with poor tumor prognosis. It can be used as a prognostic indicator for ICC tumors. Moreover, the OS and time to recurrence (TTR) of ICC patients are shortened with increasing expression levels of DKK1 (Shi et al., 2012). CYFRA21-1 is a protein fragment of cytokeratin in epithelial cells that is solubilized in the bloodstream after degradation. When the cells undergo malignant transformation, the degradation reaction is accelerated. Many cytokeratin fragments are formed and released into the bloodstream (Uenishi et al., 2003). CYFRA21-1 is active in lung, cervical, esophageal, nasopharyngeal, breast, gastrointestinal tract, bladder, and many other tumors (Zeng et al., 2018; Nakamura et al., 2019; van Rhijn et al., 2005; Teh et al., 2019). Its concentration is positively correlated with the tumor volume, degree of tumor infiltration, and clinical stage of the tumor (Uenishi et al., 2007). It is of great value in the early diagnosis of tumors, monitoring treatment efficacy, and determining patient prognosis. The optimal threshold value of serum CYFRA21-1 in ICC patients was 2.7 ng/mL (sensitivity, 74.7%; specificity, 92.2%), and this marker is highly sensitive and specific for diagnosing ICC (Uenishi et al., 2007). A high serum CYFRA21-1 concentration can predict tumor progression and poor postoperative outcomes in patients and is positively correlated with poor prognosis in patients with ICC tumors. In conclusion, DKK1 is limited to the assessment of ICC and cannot be used as a molecular marker for assessing other types of cholangiocarcinoma in addition to ICC. CYFRA21-1 is expressed across cancers and may not be used as a specific diagnostic indicator for CCA.

### 3.2 Cholangiocarcinoma and mutant genes

**FIGURE 2:**
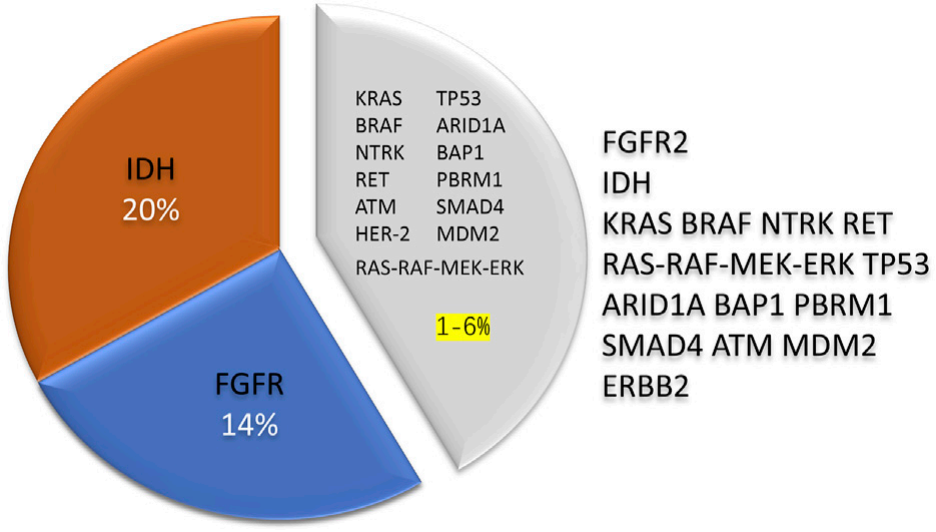
The rate of genetic variants in cholangiocarcinoma is about 40%, and IDH and FGFR2 are the most prevalent variant genes, accounting for 30%–35% of the total. In contrast, other rare genetic alterations exist in 1%–6% of the cases. Compared with different types of cholangiocarcinoma, intrahepatic cholangiocarcinoma is more likely to have genetic variations. IDH, Isocitrate dehydrogenase; FGFR, Fibroblast growth factor receptors.

Genetic variants play crucial roles in biliary tract cancer (BTC) development. Approximately 40% of BTC patients harbor genetic alterations that affect various pathways, such as cell cycle regulation, epigenetic modification, tyrosine kinase receptors, cellular signaling cascades, metabolic pathways, DNA synthesis, the DNA damage response (DDR), and homologous recombination pathways, among others (Harding et al., 2023). The genes most commonly mutated in cholangiocarcinoma (CCA) include IDH, TP53, ARID1A, BAP1, KRAS, PBRM1, BRAF, SMAD4, and ATM (Banales et al., 2020; Pirozzi and Yan, 2021; Kelley et al., 2020; Jiao et al., 2013). Potentially oncogenic copy number changes in genes include CDKN2A deletion and MDM2, ERBB2, and MCL1 amplification; gene fusions are highlighted in FGFR2 (18%) (Lowery et al., 2018; Boerner et al., 2021). Drugs that target FGFR and IDH gene variants have made significant breakthroughs in treating cholangiocarcinoma (CCA) in recent years (Pirozzi and Yan, 2021; Lee et al., 2021; Vogel et al., 2023). Fibroblast growth factor receptors (FGFRs) are a group of transmembrane tyrosine kinase receptors involved in cellular proliferation and function. They activate downstream signaling pathways by binding to ligands. These pathways regulate cell proliferation, migration, and antiapoptotic signaling (Dieci et al., 2013). Lipika et al. (Goyal et al., 2021) found that FGFR2 gene aberrations accounted for approximately 6.1% of the CCA tumors and 10%–15% of the ICC tumors studied and were associated with FGFR2 gene fusions (14%); in contrast, gene alterations were virtually absent in the ECC patients (Chmiel et al., 2022). Several studies have shown that FGFR inhibitors may be a therapeutic option for locally advanced and metastatic CCA (Goyal et al., 2021; Lee et al., 2021). Isocitrate dehydrogenase isoform (IDH) is the most commonly mutated metabolic gene in human cancers and epigenetic disorders; aberrant gene expression, blockade of differentiation, and altered metabolism can lead to the transformation of IDH-mutated cells (Pirozzi and Yan, 2021). IDH is classified into IDH1 and IDH2, which are located in the cytoplasm and mitochondria, respectively, and IDH has a 20% positive mutation rate in ICC (Pirozzi and Yan, 2021). Mutations in this gene promote immune evasion and tumor maintenance in cholangiocarcinoma (Wu et al., 2022). However, no studies have shown that FGFR and IDH gene alterations can be prognostic indicators of CCA (Figure 2).

Combining drug therapies that target FGFR and IDH gene variants appears to be beneficial for intrahepatic cholangiocarcinoma (ICC) (Pirozzi and Yan, 2021; Moris et al., 2022; Vogel et al., 2023). However, these gene variants are rare in other types of cholangiocarcinoma. Additionally, only approximately 20% of cholangiocarcinoma patients are eligible for targeted drug therapy (Pirozzi and Yan, 2021), and the rate of single gene mutation is low. This low eligibility presents a challenging situation for targeted therapy in cholangiocarcinoma. The current research status indicates that both the diagnosis and treatment of cholangiocarcinoma are pretty tricky. Therefore, emphasizing the importance of molecular analysis for cholangiocarcinoma patients and exploring the biological basis of cholangiocarcinogenesis is essential. These efforts can potentially lead to breakthroughs in diagnostic and therapeutic approaches. Further exploration into novel, specific molecular biomarkers for the diagnosis and/or treatment of cholangiocarcinoma tumors is critical.

## 4 Association of cholangiocarcinoma with Claudin 18 (CLDN18)

The protein claudin18 is not typically found in healthy bile duct tissue, but it is found at high levels in cholangiocarcinoma, a type of cancer (Takasawa et al., 2017). A study by Aya Shinozaki and colleagues used immunohistochemistry to analyze cholangiocarcinoma. They reported that approximately 43% of patients with intrahepatic cholangiocarcinoma tested positive for claudin18 expression. Moreover, more than 80% of patients with extrahepatic cholangiocarcinoma (90%), intrahepatic IPNBs (100%), and extrahepatic IPNBs (89%) also presented positive claudin18 expression (Shinozaki et al., 2011). Research has demonstrated that the protein expression of CLDN18, a protein associated with bile duct tumors, is triggered by the epidermal growth factor receptor/extracellular signal-regulated kinase (EGFR/ERK) signaling pathway. EGFR amplification and ERK activation increase CLDN18 expression (Takasawa et al., 2017), creating a feedback loop that promotes tumor development. Furthermore, RAS oncogene overexpression can induce ERK activation, further increasing CLDN18 expression. *In vitro* studies have revealed that CLDN18 promotes the growth and invasion of cholangiocarcinoma cancer cells while inhibiting CLDN18 expression significantly reduces tumorigenesis, tumor development, and tumor invasiveness (Takasawa et al., 2017).

## 5 The predictive value of CLDN18.2 in tumor-targeted drug therapy

**FIGURE 3:**
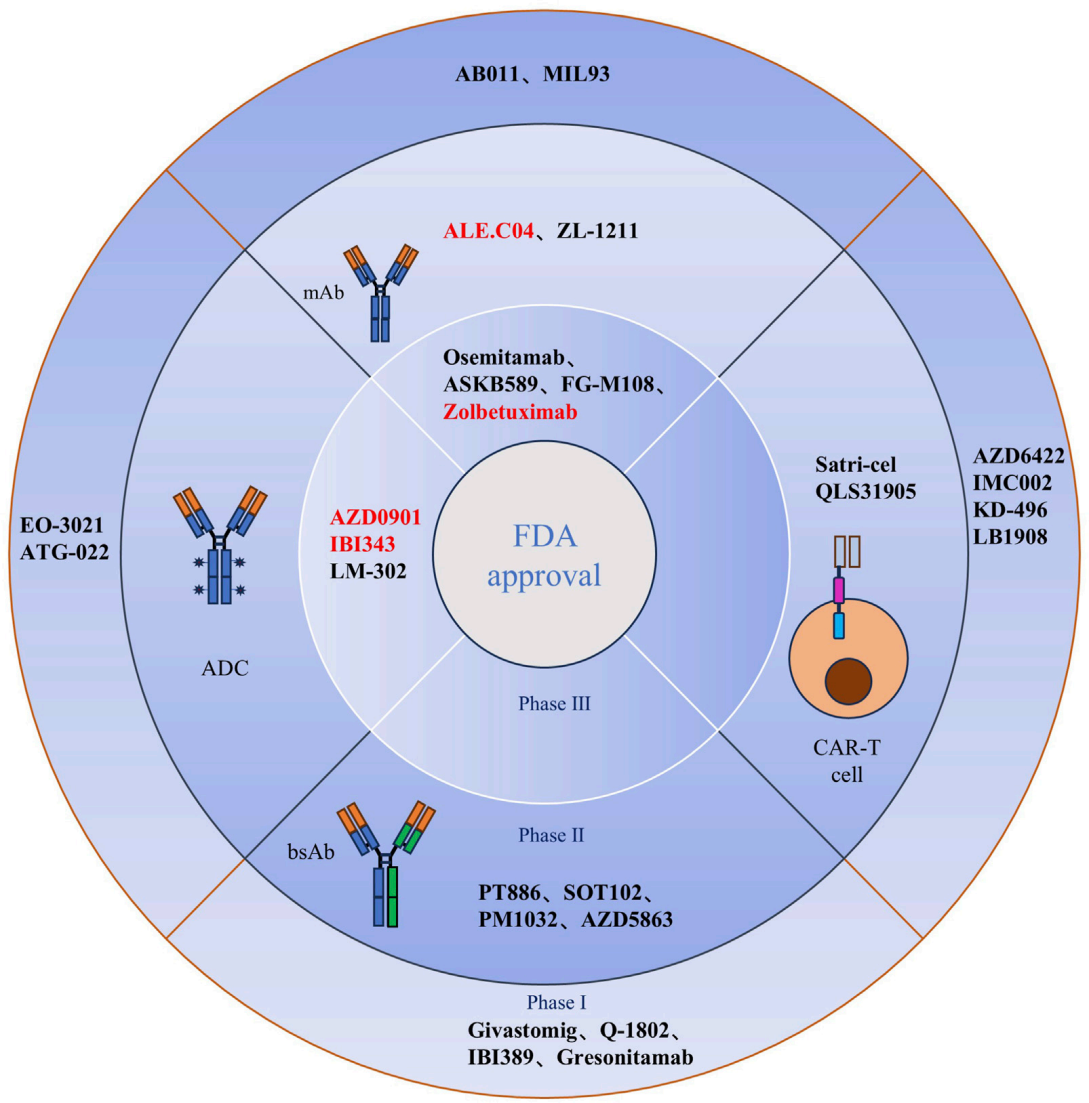
Clinical CLDN18.2-targeted therapy for cancer. A total of 26 drugs based on mAbs, bsAbs, CAR-T cell, and ADCs modalities are currently being assessed in clinical trials for the treatment of cancer. Anti-CLDN18.2 (mAbs, zolbetuximab, osemitamab, ASKB589, and FG-M108; ADCs, AZD0901, IBI343, and LM-302) have been tested in phase III clinical trials. BsAbs and CAR-T cells targeting CLDN18.2 are in phase I and/or II clinical trials. Red denotes fast-track designation or priority review designation by the US FDA. mAb, monoclonal antibody; bsAb, bispecific antibody; CAR, chimeric antigen receptor; ADC, antibody-drug conjugate; CLDN18.2, claudin18.2; Satri-cel, satricabtagene autoleucel; FDA, Food and Drug Administration.

Clinical trial studies on drug therapies that target CLDN18.2 have made significant progress in recent years. These studies focused on four major classes of drugs: monoclonal antibodies, CAR-T-cell therapy, antibody-drug conjugates (ADCs), and dual-antibody drugs. Monoclonal antibodies and CAR-T-cell therapy have emerged as the most common treatments for CLDN18.2-positive cancers, and they are currently in the late stage of Phase III clinical studies. Research has shown that innovative drugs targeting CLDN18.2 combined with first-line chemotherapy significantly improve progression-free survival (PFS) and overall survival (OS) compared with first-line chemotherapeutic agents alone. These improvements were particularly notable in patients with locally advanced or metastatic CLDN18.2-positive disease. The clinical efficacy was especially significant in patients with moderate to equivocal solid expression of CLDN18.2 in tumor cells, with sustained clinical benefits observed in more than 70% of patients. These findings suggest that CLDN18.2 holds promise as a potential therapeutic target (Several emerging drugs and CAR-T-cell drugs targeting CLDN18.2 have been developed in China, and their research stages and other information are shown in Table 2; Figure 3).

**TABLE 2:** Clinical development of CLDN18.2-targeting therapeutics.

Target/modality	Drug	Phase	Clinical trial ID	Design	Status
mAb	Zolbetuximab ^a^	III	NCT03504397	Combo	Active NR
	Osemitamab ^b^	III	NCT06093425	Combo	NyR
	ASKB589	III	NCT06206733	Combo	Recruiting
	FG-M108	III	NCT06177041	Combo	Recruiting
	ZL-1211	I/II	NCT05065710	Mono	Completed
	MIL93	I	NCT04671875	Mono	Recruiting
	AB011	I	NCT04400383	Mono and combo	Completed
bsAb	PT886 ^b^	I/II	NCT05482893	Mono and combo	Recruiting
	SOT102	I/II	NCT05525286	Mono and combo	Recruiting
	Givastomig ^b^	I	NCT04900818	Mono	Recruiting
	PM1032	I/II	NCT05839106	Mono	Recruiting
	AZD5863	I/II	NCT06005493	Mono	Recruiting
	Gresonitamab	I	NCT04260191	Mono	Terminated
	IBI389	I	NCT05164458	Mono and combo	Recruiting
	Q-1802	I	NCT04856150	Mono	Recruiting
CAR-T	Satri-cel	I/II	NCT04581473	Mono	Recruiting
	QLS31905	I/II	NCT06041035	Combo	NyR
	AZD6422	I	NCT05981235	Mono	Recruiting
	IMC002	I	NCT05472857	Mono	Recruiting
	KD-496	I	NCT05583201	Mono	Recruiting
	LB1908	I	NCT05539430	Mono	Recruiting
ADC	AZD0901 ^c^	III	NCT06346392	Mono	Recruiting
	LM-302	III	NCT06351020	Mono	Recruiting
	IBI343 ^c^	III	NCT06238843	Mono	Recruiting
	EO-3021	I	NCT05980416	Mono	Recruiting
	ATG-022 ^b^	I	NCT05718895	Mono	Recruiting

^a^ FDA, priority review, rejected owing to unspecified deficiencies in a third-party manufacturing facility, but resubmitted. mAb, monoclonal antibody; bsAb, bispecific antibody; CAR-T, chimeric antigen receptor T cell; ADC, antibody-drug conjugate; combo, combination therapy; mono, monotherapy; FDA, food and drug administration; NCT, national clinical trial; NK, natural killer; NR, not recruiting; NyR, not yet recruiting; Satri-cel, satricabtagene autoleucel.

^b^ FDA orphan drug.

^c^ FDA fast track.

### 5.1 Zolbetuximab

Zolbetuximab (IMAB362) is the first monoclonal antibody of the immunoglobulin G1 class that targets CLDN18.2 (Cao et al., 2022; van Laarhoven and Derks, 2023). This antibody specifically recognizes and binds to the Claudin18.2 protein. This highly selective antibody can trigger cytotoxicity and complement-dependent cytotoxicity against CLDN18.2-positive adenocarcinoma cells (Shah et al., 2023). The specific targeting of CLDN18.2 allows Zolbetuximab to effectively combat cancer while minimizing toxic side effects on normal tissues, resulting in high sensitivity and low toxicity advantages. According to the latest clinical trial studies, Zolbetuximab has shown significant efficacy in treating patients with CLDN18.2-positive advanced gastric adenocarcinoma. The Phase III SPOTLIGHT (NCT03504397) and GLOW (NCT03653507) studies (Shah et al., 2023; Nakayama et al., 2024), along with the Phase II MONO (NCT01197885) and FAST (NCT01630083) studies, demonstrated clinical benefit in patients with advanced gastric adenocarcinoma/gastroesophageal junction adenocarcinoma and esophageal cancer expressing CLDN18.2 (Sahin et al., 2021; Türeci et al., 2019). These findings suggest that CLDN18.2 could be a potential target for treating a wide range of solid tumors [Various clinical trials involving zolbetuximab (IMAB362) are shown in Table 3].

**TABLE 3:** Various clinical trials involving zolbetuximab (IMAB362).

Study-completion date	NCT number	Phase	Number of patients	Design	Response rate	OS	Median FPS	Adverse events
MONO-2019	NCT01197885	II	54	Single arm, repeated dose, monotherapy study	ORR-14%	_	14.5 weeks	Nausea, vomiting
FAST-2021	NCT01630083	II	84 + 77 + 85	EOX vs. Zolbetuximab (800/600 mg/㎡)+EOX vs. Zolbetuximab (1,000 mg/㎡)+EOX	ORR, Objective Response Rate-14% (39% vs. 25%)	_	5.3 vs. 7.5 months vs. -	Nausea, vomiting, decreased neutrophil count, neutropenia.
SPOTLIGHT-2023	NCT03504397	III	283 + 282	Randomized Zolbetuximab + Capox vs. Placebo + mFOLFOX6	Zolbetuximab treatment showed a significant reduction in the risk of death *versus* placebo (HR 0·75, 95% CI 0 · 60–0·94; p = 0·0053).	_	10.61 vs. 8.67 months	Nausea, vomiting, decreased appetite
GLOW-2023	NCT03653507	III	253 + 250	Randomized Zolbetuximab + Capox vs. Placebo + Capox	ORR-5%	14.39 vs. 12.16 months	8.2 vs. 6.8 months	Nausea, vomiting

SPOTLIGHT (NCT03504397), a global Phase III study involving patients with CLDN18.2-positive tumors (≥75% of tumor cells have moderate to strong claudin-18.2 (CLDN18.2) membrane staining) and human epidermal growth factor receptor 2 (HER2)-negative disease (Shah et al., 2023), recently reported preliminary results showing that patients receiving Zolbetuximab plus mFOLFOX6 (modified fluorouracil, fluorouracil, and oxaliplatin regimen) showed a significant benefit in terms of progression-free survival (PFS) and median PFS in patients treated with Zolbetuximab plus mFOLFOX6 *versus* placebo plus mFOLFOX6 (median PFS: 10.61 months vs 8.67 months, hazard ratio (HR) = 0.75; PFS: 12.94 months vs 12.65 months) (Shah et al., 2023; Lordick et al., 2024; Kohei et al., 2023). In the GLOW (NCT03653507) study, zolbetuximab, a monoclonal antibody against CLDN18.2, in combination with capecitabine and oxaliplatin (CAPOX) as a first-line treatment for CLDN18.2-positive, HER2-negative, locally advanced unresectable or metastatic GC/GEJ adenocarcinoma significantly prolonged patients’ PFS and OS (overall survival) (Shah et al., 2023). Survival was prolonged considerably in GC/GEJ adenocarcinoma patients treated with Zolbetuximab plus CAPOX compared with those treated with placebo plus CAPOX (median PFS: 8.2 months vs 6.80 months, HR = 0.687; median OS: 14.4 months vs 12.2 months, HR = 0.771) (Shah et al., 2023). The most common Grade ≥3 treatment-emergent adverse events (TEAEs) that occurred in the SPOTLIGHT and GLOW studies were nausea, vomiting, and loss of appetite, and nausea and vomiting could be controlled with antiemetic medications, interruption of drugs, and adjustments to the infusion rate (Shah et al., 2023; Kohei et al., 2023). The most common TEAEs were nausea and vomiting.

A benefit was first observed among patients with CLDN18.2-positive advanced esophageal cancer treated with Zolbetuximab in Phase II MONO study 57 and confirmed in Phase II FAST study (Sahin et al., 2021). Compared with patients treated with the etoposide + oxaliplatin + capecitabine (EOX) regimen alone, patients with CLDN18.2-positive advanced gastric adenocarcinoma, gastroesophageal junction adenocarcinoma and esophageal adenocarcinoma treated with EOX + Zolbetuximab, patients with CLDN18.2-positive advanced gastric adenocarcinoma, gastroesophageal junction adenocarcinoma, and esophageal adenocarcinoma treated with EOX + Zolbetuximab showed significant improvements in PFS and OS [hazard ratio (HR) = 0.44]. The PFS benefit was maintained in patients with high CLDN18.2 expression (≥70% of the tumor cells showed moderate to solid CLDN18.2 expression) (HR = 0.38) (Sahin et al., 2021). These two studies reported the same grade 3 or higher TEAE of nausea and vomiting. Through Phase III clinical studies, we learned that treatment-related TEAEs of Grade 3 or higher from zolbetuximab can be effectively controlled with symptomatic management and are well within acceptable limits regarding its efficacy.

### 5.2 Bispecific antibodies (BsAbs)

Bispecific antibodies (BsAbs) are artificial antibodies that simultaneously bind to two antigens or antigenic epitopes. BsAb can target different antigens or epitopes on the surface of tumor cells, leading to anti-tumor effects. They can also block multiple signaling pathways in tumor development and recruit T-cells or natural killer cells (Labrijn et al., 2019). One specific example is the PD-L1/Claudin18.2 (CLDN18.2) bispecific antibody, a research hotspot. This antibody can directly kill tumor cells via CLDN18.2 antibody-mediated ADCC/CDC while simultaneously blocking the PD-1/PD-L1 signaling pathway to activate an adaptive anti-tumor immune response (Zhu et al., 2020). SPX301 is the first PD-L1/CLDN18.2 antibody to be used globally. Preliminary results of CLDN18.2 bispecific antibody in preclinical trials show that it can effectively inhibit the growth of CLDN18.2-positive MC38 tumors with low immunogenicity and a good safety profile (Zhu et al., 2020). China’s independently developed PD-L1/CLDN18.2 bispecific antibody Q-1802 is the first of its kind to be approved for clinical trials by the Food and Drug Administration (FDA) of the United States. A phase I clinical trial of IBI389, a bispecific antibody that targets CLDN18.2/CD3, is currently underway to assess the efficacy of BsAb in CLDN18.2-positive refractory advanced solid tumors (Zhou et al., 2024). In a study of 26 patients with advanced gastric adenocarcinoma/gastroesophageal junction adenocarcinoma, it was found that those with moderate to isoform solid expression of CLDN18.2 in ≥10% of the tumor cells had an overall response rate (ORR) of 30.8%, a disease control rate (DCR) of 73.1%, and median progression-free survival (PFS) of 3.5 months (Li et al., 2024). Additionally, 60% of these patients experienced grade 1-2 cytokine release syndrome (CRS) (Li et al., 2024). In another group of 27 patients with advanced pancreatic ductal adenocarcinoma, where ≥10% of tumor cells showed moderate to formal solid expression of CLDN18.2, the ORR was 29.6%, and the DCR was 70.4% (Zhou et al., 2024).

For cold tumors that are poorly immunogenic and have a negligible response to immune checkpoint blockade therapies, unique treatments are required to promote recognition, presentation, and phagocytosis of tumor cells by immune cells. Zhang et al. (2024) genetically engineered two single-stranded variable fragments (scFvs) on cell membranes: a scFv targeting CLDN18.2 for the specific recognition of tumor cells and a scFv targeting CD40 for binding to macrophages. Immunotherapy of highly aggressive cold tumors was achieved by introducing a bispecific antibody-based nano capture that combined the two antibody-anchored membranes with genetic engineering (Zhang et al., 2024). This nanotechnology developed by Zhang et al. significantly enhanced the anti-tumor efficacy of highly aggressive cold pancreatic cancers (Zhang et al., 2024). The significant enhancement of the immune effect is achieved by increasing the recognition and phagocytosis of tumor cells by macrophage cells, enhancing activation and antigen presentation, and increasing the activity of cytotoxic T-lymphocytes (Zhang et al., 2024). The BsAb analogs utilized to treat CLDN18.2-positive advanced refractory solid tumors display great potential. Combined with nanotechnology, they enhance the precision and efficiency of anti-tumor therapy.

### 5.3 CAR-T cells

A Phase I clinical trial of high-affinity humanized CLDN18.2 autologous chimeric antigen receptor (CAR)-T cells [Satricabtagene autoleucel (Satri-cel)/CT041] specifically targeting CLDN18.2, the CT041--CG4006 trial (NCT03874897), has been completed (Qi et al., 2022). Interim results showed acceptable safety and efficacy in CLDN18.2-positive advanced gastric adenocarcinoma patients who were followed up in the clinical trial (Qi et al., 2022). The final results revealed that satri-cel treatment of patients with CLDN18.2-positive advanced gastrointestinal cancer is safe and effective (Qi et al., 2024). Based on the promising safety and efficacy of CT041 in the CT041-CG4006 trial (NCT03874897) (Qi et al., 2024), a multicenter randomized Phase 2 trial of CT041/Satri-cel (NCT04581473), a CAR-T-cell therapy specific for CLDN18.2, is underway (Qi et al., 2024). This trial is a global clinical trial of CAR-T-cell therapy for the treatment of solid tumors, and the results from a long-term follow-up trial demonstrated that sari-cel/CT041 did not cause dose-limiting toxicity, immune effector cell-associated neurotoxicity syndrome (ICANS), hemophagocytic lymphohistiocytic hyperplasia (HLH), or treatment-related death (Qi et al., 2024). The common adverse reactions associated with CAR-T-cell therapy included Grade 3/4 transient hematological toxicity (100%) and Grade 1/2 cytokine release syndrome (95%) due to clearing preconditioning treatment. These reactions may be linked to the fully humanized scFv fragment and the clearing preconditioning regimen (Feng et al., 2017; Liu et al., 2020). Compared with zolbetuximab, CAR-T-cell therapy resulted in a lower incidence of Grade 3 or higher treatment-related vomiting adverse events (3.1% vs 22%) (Qi et al., 2024).

Overall, CAR-T-cell therapy has a manageable safety profile and significant efficacy (Qi et al., 2022). Compared with previous CAR-T-cell therapy for solid tumors, the safety profile of CT041 infusion and the copy number and persistence of CT041 were improved.

### 5.4 Antibody‒drug coupling (ADC)

ADCs consist of monoclonal antibodies, linkers, and cytotoxic drugs with high specificity and antitumor activity. Since the first ADC drug, gemtuzumab, was approved by the FDA in 2000 (O’Brien et al., 2023), ADCs have demonstrated good antitumor activity and development prospects. CMG-901 is the most recent ADC to target CLDN18.2 and is currently in Phase I clinical trial (NCT04805307). It delivers the antimitotic agent monomethyl auristatin E (MMAE) into tumor cells by binding to CLDN18.2 to kill tumor cells. Another anti-CLDN18.2 ADC drug, SYSA1801 injection, was approved for clinical trials to treat advanced solid tumors by the Drug Review Center of the State Drug Administration of the People’s Republic of China in June 2021.

Various innovative drugs, including monoclonal antibodies, bispecific antibodies, and antibody‒drug couplings (ADCs) (Nakayama et al., 2024), that target CLDN18.2 are continuously being developed. Compared with traditional antitumor approaches, emerging innovative drugs may significantly impact the treatment landscape for GI malignancies and drive innovative research with better prospects for a broader scope of application.

## 6 Conclusion: potential correlation between CLDN18.2 and cholangiocarcinoma and its application prospects

CLDN18.2, a subtype of the CLDN18 gene, exhibits abnormal expression across numerous solid tumors, particularly in gastrointestinal adenocarcinomas such as gastric adenocarcinoma, gastroesophageal junction adenocarcinoma, and esophageal adenocarcinoma. Compared to its counterpart, CLDN18.1, CLDN18.2 is more prone to abnormal cholangiocarcinoma expression, including adenocarcinomas in over 90% of cases. Research has demonstrated that CLDN18 is overexpressed in cholangiocarcinoma, promoting tumorigenesis, cellular proliferation, and invasiveness. Moreover, the exposure of CLDN18.2 epitopes is typically associated with the disruption of tight junctions resulting from epithelial cell carcinogenesis. This suggests that the overexpression of CLDN18.2 may be intrinsically linked to the initiation, progression, and distant metastasis of cholangiocarcinoma cells, potentially serving as an independent prognostic indicator of poor outcomes. CLDN18 is exclusively expressed in cholangiocarcinoma tissues and absent in normal biliary epithelium, making CLDN18.2 a tumor-specific biomarker that effectively differentiates normal biliary epithelium from cholangiocarcinoma.

In light of the generally poor prognosis associated with cholangiocarcinoma and the limited efficacy of existing therapeutic modalities, exploring novel therapeutic targets becomes essential. Emerging therapeutic agents targeting CLDN18.2—including monoclonal antibodies, bispecific antibodies, CAR-T cell therapies, and ADCs—have shown promising efficacy in clinical settings. These therapeutic strategies successfully inhibit the proliferation and metastasis of tumor cells by explicitly targeting CLDN18.2, thereby improving both survival rates and quality of life for patients. Notably, the incidence of grade ≥3 drug-related adverse events remains relatively low and manageable with these treatments, presenting them as safer alternatives compared to traditional chemotherapy for patients expressing CLDN18.2.

Furthermore, the rapid advancement of nanotechnology has opened avenues for targeted nanomedicines focused on CLDN18.2, providing innovative therapeutic options for patients. Utilizing nanocarriers to deliver drugs targeting CLDN18.2 precisely can significantly enhance drug bioavailability while reducing systemic side effects, ultimately improving patients’ quality of life. Implementing nanotechnology enables a more flexible and diverse range of treatment strategies targeting CLDN18.2, fostering synergistic interactions with chemotherapy and immunotherapy, thereby increasing overall treatment efficacy. Future research should prioritize exploring CLDN18.2’s potential applications within various therapeutic frameworks, particularly in personalized medicine and combination treatment strategies. Developing intelligent nanoparticles and multifunctional nanocarriers will further strengthen the synergy between chemotherapy, immunotherapy, and targeted therapies, enhancing treatment precision and effectiveness.

Although ongoing investigations into CLDN18.2 remain in the clinical trial phase, its potential as a therapeutic target for CLDN18.2-positive tumors is undeniably promising. Integrating targeted therapies and nanomedicine approaches centered on CLDN18.2 ushers in a new era of hope for cholangiocarcinoma patients. Future studies must continue to refine these treatment strategies to achieve superior clinical outcomes. Looking ahead, the potential applications of CLDN18.2 in cholangiocarcinoma spark significant anticipation, as they may introduce new paradigms in treating this challenging disease. Ultimately, nanotechnology and targeted therapies based on CLDN18.2 are poised to play central roles in cholangiocarcinoma treatment, transforming therapeutic modalities and significantly enhancing patients’ survival and quality of life. Nevertheless, the specific roles and expression levels of CLDN18.2 in cholangiocarcinoma require further investigation and validation to deepen our understanding of its relationship with the disease and its clinical significance. Through continued research and clinical trials, CLDN18.2 could emerge as a pivotal breakthrough in the treatment landscape of cholangiocarcinoma, offering patients more effective therapeutic options and improved quality of life.
